# The Role of Systemic Inflammatory Indices in Predicting Cardiovascular Involvement in Children with Duchenne Muscular Dystrophy

**DOI:** 10.3390/children12091164

**Published:** 2025-09-01

**Authors:** Ecem İpek Altınok, Taner Kasar

**Affiliations:** 1 Department of Pediatrics, Faculty of Medicine, Ordu University, 52200 Ordu, Türkiye; 2 Division of Pediatric Cardiology, Faculty of Medicine, Ordu University, 52200 Ordu, Türkiye; tanerkasar@odu.edu.tr

**Keywords:** Duchenne Muscular Dystrophy, cardiomyopathy, electrocardiography, echocardiography, inflammatory indices, Pro-BNP

## Abstract

**Background:** Duchenne Muscular Dystrophy (DMD) is an X-linked recessive neuromuscular disorder that is characterized by progressive muscle weakness, musculoskeletal limitations, and pulmonary involvement, with cardiomyopathy and cardiovascular complications being a primary cause of morbidity and mortality. With advances in respiratory care, cardiac involvement has become the leading cause of death. There is growing interest in systemic inflammatory indices as potential predictors of cardiovascular involvement. This study aimed to evaluate the prognostic value of inflammatory markers—neutrophil-to-lymphocyte ratio (NLR), monocyte-to-lymphocyte ratio (MLR), platelet-to-lymphocyte ratio (PLR), systemic inflammatory response index (SIRI), systemic immune-inflammation index (SII), and pan-immune inflammation value (PIV)—in children with DMD and to explore their association with cardiac findings. **Methods:** In this retrospective study, 25 male patients diagnosed with DMD and 25 age-matched healthy male controls were evaluated between January 2021 and July 2024. Demographic and clinical data, hematologic and biochemical parameters, and inflammatory indices were recorded. Cardiovascular involvement was assessed using electrocardiography (ECG) and transthoracic echocardiography (TTE). Group comparisons were performed using independent *t*-tests, while ROC and Pearson correlation analyses were used for diagnostic performance and associations. **Results:** Pathological Q waves were the most frequent ECG abnormality (24%), and 16% of patients had echocardiographic abnormalities. While most systemic inflammatory indices (NLR, MLR, SIRI, SII, PIV) did not significantly distinguish cardiovascular involvement, PLR demonstrated a strong positive correlation with Pro-BNP levels (r = 0.86, *p* < 0.05), suggesting a potential link between systemic inflammation and subclinical cardiac stress. **Conclusions:** Although the overall diagnostic utility of inflammatory indices in predicting cardiovascular complications in DMD was limited, PLR showed a correlation with Pro-BNP in our cohort. However, given the small sample size and limited number of patients with ventricular dysfunction, this finding should be interpreted with caution. PLR may warrant further investigation as a potential marker of cardiovascular involvement in DMD, but larger prospective studies are needed to validate its clinical significance.

## 1. Introduction

Duchenne Muscular Dystrophy (DMD) is an X-linked inherited disorder characterized by progressive muscle weakness and degeneration, primarily observed in male children. Epidemiologically, DMD is one of the most common neuromuscular disorders of childhood, with an estimated incidence of approximately 1 in 3500–5000 live male births worldwide, corresponding to a prevalence of about 1 in 10,000–20,000 boys [1,2,3]. The etiology of DMD is linked to a genetic defect resulting in absent or dysfunctional production of the protein dystrophin. This deficiency leads to muscle fiber damage and subsequent loss of muscle function. However, DMD is not limited to skeletal muscle involvement; its systemic effects also contribute to impairment of the cardiovascular system. While both skeletal and cardiac dysfunction worsen over time, their progression is often not parallel; some patients with advanced skeletal muscle weakness may have relatively preserved cardiac function, whereas others may develop significant cardiac involvement even in earlier disease stages [4].

Cardiovascular involvement is a frequently overlooked but critical component of DMD. Most children with DMD develop cardiac muscle weakness (cardiomyopathy) that becomes more pronounced with advancing age. This cardiomyopathy mainly manifests as left ventricular dysfunction, which can eventually progress to heart failure [5]. Additionally, arrhythmias and other electrophysiological abnormalities play a significant role in disease progression and contribute to increased mortality rates in these patients [6].

Non-invasive diagnostic tools such as electrocardiography (ECG) enable early detection of cardiovascular involvement in DMD. ECG provides valuable information on cardiac status; commonly observed abnormalities in children with DMD include QRS complex widening, PR interval prolongation, T-wave inversions, and pathological Q waves [7].

Recently, systemic inflammatory indices have gained prominence as novel markers for predicting cardiovascular disease prognosis and quantifying inflammation. Indices such as neutrophil-to-lymphocyte ratio (NLR), monocyte-to-lymphocyte ratio (MLR), platelet-to-lymphocyte ratio (PLR), systemic inflammatory response index (SIRI: neutrophils × monocytes/lymphocytes), systemic immune-inflammation index (SII: neutrophils × platelets/lymphocytes), and pan-immune inflammation value (PIV: neutrophils × monocytes × platelets/lymphocytes) are simple, low-cost parameters derived from routine blood tests that reflect the extent of systemic inflammation. Their prognostic values have been demonstrated in various cardiovascular, neurovascular, oncological, and metabolic disorders [8,9,10].

Early identification and management of cardiovascular complications in children with Duchenne Muscular Dystrophy may improve disease prognosis. In this context, further research is required to clarify whether systemic inflammatory indices can serve as predictors of cardiovascular involvement in this population. This study aims to investigate the cardiovascular effects and electrocardiographic findings of DMD and to evaluate the potential utility of novel inflammatory indices in guiding cardiac monitoring and treatment strategies.

## 2. Materials and Methods

This retrospective study evaluated data from 25 patients diagnosed with Duchenne Muscular Dystrophy (DMD) who were under follow-up in the pediatric cardiology outpatient clinic between 1 January 2021 and 1 July 2024. All eligible patients under 18 years of age during this period were included. The diagnosis of Duchenne Muscular Dystrophy in all patients was confirmed based on clinical features, persistently elevated serum creatine kinase (CK) levels, and genetic testing demonstrating dystrophin gene mutations. Muscle biopsy was not required since molecular genetic confirmation was available for all patients. The control group consisted of an equal number of healthy male children aged 2 to 18 years, randomly selected from individuals presenting to the pediatric outpatient clinic who had undergone complete blood counts as part of their routine clinical evaluation. The number of controls was determined based on an a priori power analysis. Assuming a two-tailed α of 0.05, a power of 80%, and a large effect size (Cohen’s *d* = 0.80), the analysis indicated that a minimum of 25 participants per group would be sufficient; therefore, 25 controls were included.

The study was conducted in accordance with the Declaration of Helsinki and approved by the Non-Interventional Research Ethics Committee of Ordu University (protocol code 2025/68; date of approval: 7 March 2025). As this was a retrospective study, informed consent could not be obtained directly from the patients or from the individuals in the control group; however, all data were collected and analyzed in accordance with ethical guidelines, and patient confidentiality was strictly maintained.

The variables assessed included patient age, age at diagnosis, wheelchair dependence, tracheostomy status, treatment regimens, hematological parameters, NLR, MLR, PLR, SIRI, SII, PIV, ECG, and ECHO parameters. Complete blood count and biochemical parameters were obtained as part of the patients’ routine clinical follow-up visits in the pediatric cardiology outpatient clinic. These assessments were performed at the time of presentation for regular monitoring, and not during acute illness or intercurrent infection. Additional metrics, including Pro-BNP, ECG, and ECHO, were collected as part of the standard cardiological evaluation protocol for patients with DMD. In our center, ECG is typically performed at each annual follow-up visit to monitor electrical abnormalities such as conduction defects or arrhythmias, while echocardiography is performed annually or more frequently if clinically indicated, in line with current recommendations for the surveillance of cardiac function in DMD patients.

### Statistical Analysis

All statistical analyses were performed using SPSS (Statistical Package for the Social Sciences) for Windows, version 26 (IBM Corp., Armonk, NY, USA), with statistical significance set at *p* < 0.05. The normality of continuous variables was assessed using the Kolmogorov–Smirnov test and skewness–kurtosis measures. Since the data were normally distributed, parametric tests were applied.

Descriptive statistics were presented as mean, standard deviation, count (n), and percentage (%). Independent samples *t*-test was used for comparisons between groups. Receiver operating characteristic (ROC) curve analysis was conducted to determine optimal cut-off values for variables within the patient group, calculating area under the curve (AUC), sensitivity, and specificity.

Pearson correlation coefficient was employed to evaluate relationships between continuous variables.

## 3. Results

### 3.1. Demographic and Clinical Characteristics

In this retrospective study, clinical data from 25 patients diagnosed with Duchenne Muscular Dystrophy (DMD) were analyzed. All patients were male. The median age was 12 years (range: 5–18), with a mean age of 11.2 ± 4.3 years. The mean age at diagnosis was 4.8 ± 2.6 years, with a median age of 4 years (range: 2–10 years). Wheelchair dependence was observed in 16% of the patients. None of the patients required home mechanical ventilation or tracheostomy during the study period.

Regarding baseline medication regimens, all patients with DMD were receiving corticosteroid therapy. Cardiac medications were prescribed only to a minority of patients. Of these, one patient was treated with an angiotensin-converting enzyme (ACE) inhibitor, one with a combination of beta-blocker and ACE inhibitor, and one with digoxin, ACE inhibitor + beta blocker. Descriptive statistics for other measured parameters are presented in Table 1.

### 3.2. Electrocardiographic and Echocardiographic Findings

**Figure 1:**
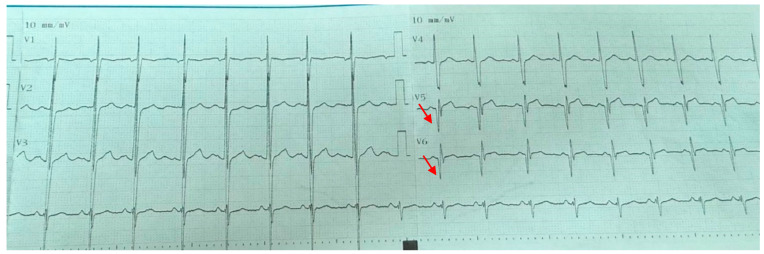
Electrocardiogram demonstrating pathological Q waves approximately 5 mm in depth in leads V5 and V6 (indicated by arrows).

**Figure 2:**
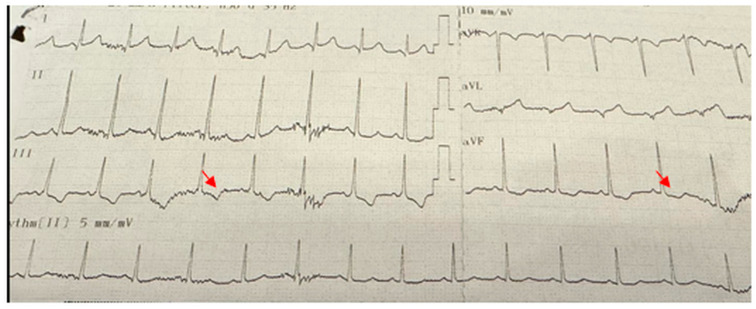
Electrocardiogram demonstrating T-wave inversion in the inferior leads (III and aVF), indicated by arrows.

Electrocardiographic abnormalities were detected in 9 (36%) of the patients. The most common ECG abnormality was pathological Q waves observed in the V5–V6 leads in 6 patients (24%) (Figure 1). Other abnormalities included T-wave inversion in the inferior leads in one patient (Figure 2), right bundle branch block in another, and supraventricular tachycardia in a third patient. Among these 9 patients with ECG abnormalities, transthoracic echocardiography (TTE) was normal in 6 (66.7%). Of the remaining three patients, one had left ventricular systolic dysfunction, another presented with both left ventricular dysfunction and dilated cardiomyopathy, and the third was diagnosed with mitral valve prolapse accompanied by mitral regurgitation. All three patients who received cardiac treatment were among those with echocardiographic abnormalities. Two of these patients exhibited pathological Q waves on ECG, while one patient (25%) showed T-wave inversion. Echocardiographic and electrocardiographic abnormalities of the patients are summarized in Table 2.

When comparing hematological parameters, hemoglobin levels were found to be significantly higher in patients with DMD compared to the control group. The mean hemoglobin level in the DMD group was 13.46 ± 1.11 g/dL, whereas in the control group it was 12.65 ± 0.84 g/dL. This difference between the two groups was statistically significant (*p* = 0.006), indicating a notable increase in hemoglobin levels in DMD patients relative to controls.

No statistically significant differences were observed between groups for other hematological parameters. Although white blood cell (WBC), lymphocyte, neutrophil, monocyte, and platelet (PLT) counts appeared slightly elevated in the patient group compared to controls, these differences did not reach statistical significance (Table 3).

### 3.3. Inflammatory Marker Levels

Key inflammatory markers were examined in patients with Duchenne Muscular Dystrophy. The mean NLR was 2.00 ± 2.28, showing high inter-individual variability. Platelet-to-lymphocyte ratio was measured at 102.63 ± 46.92, while MLR was low at 0.21 ± 0.15. The systemic inflammatory response index and SII demonstrated considerable variation within the patient group, with mean values of 0.14 ± 0.19 and 592.8 ± 659.16, respectively. The pan-immune inflammation value was 502.84 ± 103.66.

Comparison of these inflammatory markers between the patient and control groups revealed no statistically significant differences for NLR, PLR, MLR, SIRI, SII, and PIV. The results are presented in Table 4.

### 3.4. Correlation Analysis

Correlation analysis was performed among the measured parameters within the patient group. Among the cardiac markers, Pro-BNP was measured in 5 patients, and a statistically significant positive correlation was identified between Pro-BNP levels and PLR values (r = 0.86, *p* < 0.05). This indicates that as Pro-BNP levels increase, PLR values also tend to increase. In contrast, no statistically significant correlations were found among other paired variables outside of those mentioned (*p* > 0.05) (Table 5).

### 3.5. ROC Analysis of Inflammatory Markers

**Figure 3:**
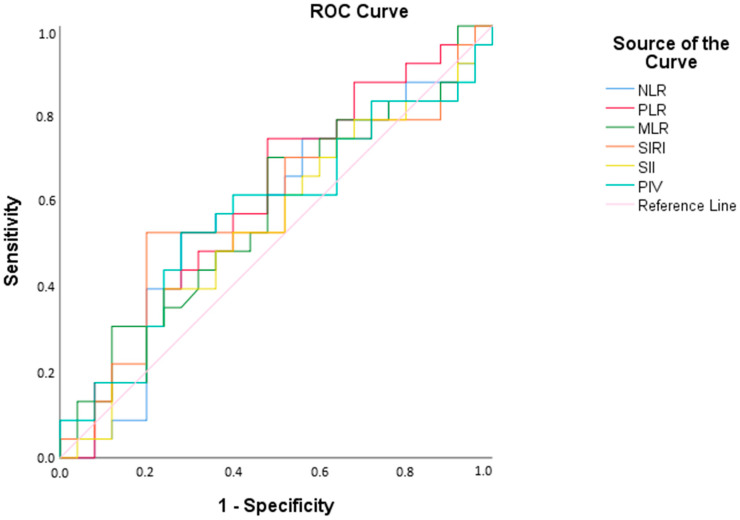
ROC Curve for IMA Measurement Across Groups.

The receiver operating characteristic analysis revealed that the NLR had a cut-off value of 1.21, with an AUC of 56.5%, indicating its discriminative ability between patient and control groups. However, based on this calculation, the sensitivity (56.5%) and specificity (60.0%) values demonstrate that the NLR measurement does not provide statistically adequate discrimination between the patient and control groups (*p* < 0.05). Similarly, PLR, MLR, SIRI, SII, and PIV also showed insufficient discriminative power between the patient and control groups (*p* < 0.05) (Figure 3, Table 6).

### 3.6. Comparison by ECG/ECHO Abnormalities

Comparisons between patients with and without ECG/ECHO abnormalities revealed no statistically significant differences in terms of age, age at diagnosis, or laboratory values (*p* > 0.05) (Table 7).

## 4. Discussion

This study examined the clinical, biochemical, and cardiological data of 25 patients diagnosed with DMD. The findings provide valuable insights into the clinical course, treatment strategies, and cardiac complications associated with DMD.

All patients included in the study were male, which is consistent with the expected inheritance pattern of DMD as an X-linked genetic disorder [1]. The median age of patients was 12 years, and the mean age at diagnosis was 4.8 ± 2.6 years. This finding emphasizes that DMD is typically diagnosed during childhood, highlighting the importance of early diagnosis in monitoring disease progression [11]. The diagnosis of DMD is commonly established between ages 2 and 5, and early diagnosis can shorten the time to treatment initiation, aiding in the preservation of motor functions [12]. In our cohort, the patient age range was 5–18 years, whereas controls were defined between 2–18 years of age to reflect the full pediatric spectrum in which DMD typically manifests and is clinically monitored. While this difference in lower age limits might raise concern for potential confounding, the median age did not differ significantly between groups, suggesting that age-related bias was unlikely to have influenced the main findings.

Regarding physical status and needs, the rate of wheelchair use was found to be 16%. The median age of our cohort (12 years) is clinically meaningful, as this period coincides with the expected onset of key disease milestones such as loss of ambulation (~13 years) [13] This indicates significant motor function decline with advancing age, with wheelchair use typically required in later stages. None of the patients required home mechanical ventilation or tracheostomy.

Cardiological assessments revealed important findings. The most frequently observed ECG abnormality was pathological Q waves (24%), consistent with literature reports indicating a 30–40% prevalence in DMD patients. This finding is considered an early marker of silent myocardial fibrosis and is associated with cardiomyopathy [14]. Another study linked frequent pathological Q waves in DMD patients with sudden cardiac death (SCD) [15].

Cardiac complications in DMD are known to commence early and become more pronounced with age [16]. Myocardial fibrosis is typically detected by cardiac MRI around the early teenage years (median age approximately 13.8 years; range 7–17 years) [17], and initial left ventricular systolic dysfunction often becomes apparent by echocardiography during mid-adolescence [18]. In our cohort, the mean age of patients with echocardiographic or electrocardiographic abnormalities was 12.1 years, reinforcing that clinically significant cardiac involvement may already be present at this stage. Pathological Q waves were identified at mean age of 12 (range 6–18 years), indicating that this abnormality may appear as early as the first decade of life and persist into late adolescence. T-wave inversion was noted at 11 years of age. Systolic left ventricular dysfunction was observed at 14 and 17 years, while dilated cardiomyopathy was diagnosed at 18 years, underscoring that severe cardiac involvement can manifest relatively early in the disease course. However, no statistically significant age difference was detected between the groups with and without cardiac pathology in our study.

The detection of severe cardiac abnormalities such as dilated cardiomyopathy on echocardiography underscores the necessity of continuous cardiological monitoring. In our cohort, echocardiographic evaluation revealed left ventricular dysfunction or structural anomalies in 16% (4/25) of patients. The weak correlation observed between echocardiographic abnormalities and ECG findings further emphasizes the limited diagnostic value of ECG as a standalone tool in DMD [19]. Moreover, cardiac MRI is recognized as the gold standard imaging modality for the early detection of cardiovascular involvement in DMD, particularly for identifying myocardial fibrosis and subclinical dysfunction [20]. In our center, cardiac MRI is available and performed in some patients; however, due to the retrospective design and lack of standardized MRI data across the cohort, these results were not included in the present analysis. Nevertheless, it should be acknowledged that while echocardiography remains the most accessible technique in routine practice, cardiac MRI provides superior sensitivity for detecting early cardiac pathology.

Beyond conventional monitoring, a potential strategy to reduce cardiovascular comorbidities, mitigate the progressive impact of DMD, and improve quality of life is to ensure normal pubertal development, which is frequently delayed due to chronic corticosteroid therapy. Although no official guidelines currently exist regarding puberty induction protocols, a recent scoping review has synthesized the available evidence, highlighting that timely pubertal induction may enhance quality of life and, indirectly, contribute to a reduction in cardiovascular risk [21].

Cardiac treatments employed included ACE inhibitors, beta blockers, and digoxin. These therapies are vital for managing cardiomyopathy and other cardiac complications, highlighting the critical role of cardiac monitoring and treatment in DMD patients [22]. The use of cardiac therapy was confined to patients with ECG or echocardiographic abnormalities, consistent with current recommendations. Nevertheless, it should also be acknowledged that some guidelines and workshop reports recommend the prophylactic initiation of ACE inhibitors by the age of 10, even in the absence of echocardiographic evidence of disease [23]. Specifically, one patient with left ventricular systolic dysfunction and pathological Q waves was treated with an ACE inhibitor in combination with a beta blocker, another patient with dilated cardiomyopathy, concomitant left ventricular systolic dysfunction, and pathological Q waves received digoxin, an ACE inhibitor+ beta blocker, and a patient who presented with supraventricular tachycardia (SVT) was managed with a beta blocker. No significant relationship was found between clinical or biochemical parameters and cardiac treatment. These findings indicate that cardiac complications should be closely monitored through ECG and echocardiography, and that a multidisciplinary approach is necessary in managing these patients [17].

When comparing hematological parameters, hemoglobin levels were significantly higher in the patient group (*p* = 0.006). Although elevated hemoglobin is uncommon in DMD, some studies report increases secondary to corticosteroid therapy [14]. However, aside from this finding, no statistically significant differences were observed between groups for the other hematological parameters.

Yükcü and Arslan demonstrated that MLR, SIRI, and PIV have acceptable diagnostic value for detecting ascending aortic dilation in children with bicuspid aortic valve [10]. In contrast, in our patients no significant differences were observed between the patient and control groups with respect to inflammatory markers, suggesting a limited role for systemic inflammatory biomarkers in the routine monitoring of DMD [24]. Nevertheless, it should be acknowledged that corticosteroid therapy, which is routinely administered in DMD, has been shown to delay the progression of cardiomyopathy, and this beneficial effect may be at least partly mediated by modulation of inflammatory pathways. Therefore, while our findings suggest a limited direct role of systemic inflammatory indices, the contribution of inflammation cannot be fully excluded.

A notable finding in our study is the observed positive correlation between Pro-BNP and PLR. However, this result should be interpreted with caution, as only a limited number of patients in our cohort had elevated NT-proBNP and ventricular dysfunction. While the association may suggest a potential link between increased cardiovascular burden and inflammatory response, the small sample size precludes definitive conclusions. Although several studies have examined the prognostic value of PLR in cardiovascular diseases, it is not considered an independent predictor of mortality. For example, a 2019 cohort study reported that higher PLR levels in patients with acute heart failure (AHF) were associated with increased mortality rates [25]. Studies directly evaluating the relationship between Pro-BNP and PLR remain limited; however, both biomarkers have been shown to be important in assessing heart failure and acute coronary syndromes. For example, a study on chronic heart failure patients found a positive correlation between NT-proBNP levels and PLR [26]. To our knowledge, no previous studies have investigated this correlation specifically in the DMD population, and further research with larger cohorts is warranted to clarify its clinical significance. However, it should be noted that PLR values in our cohort demonstrated a large standard deviation, and only a small subset of patients exhibited elevated NT-proBNP levels. Therefore, the observed correlation between PLR and NT-proBNP should be interpreted with caution. Moreover, NT-proBNP was evaluated only in the patient group, and not in the controls, which further limits the generalizability of this finding. Therefore, the significant correlation observed between PLR and NT-proBNP should be interpreted with caution, as it may represent a type I error rather than a definitive biological association. Future studies with larger cohorts and appropriate statistical corrections are required to validate this result.

The ROC analysis revealed that none of the inflammatory parameters achieved sufficient diagnostic accuracy, limiting their utility for routine screening in DMD. Furthermore, the impact of routine steroid use on inflammatory indices remains unclear and warrants further investigation. Although corticosteroids are known to impact hematological parameters—most notably by causing neutrophilia and lymphopenia, the magnitude of these changes is typically modest. Moreover, some clinical studies indicate that systemic inflammatory indices such as NLR and PLR may not be significantly altered in every context. For example, in children with steroid-sensitive nephrotic syndrome, no significant differences in PLR were observed before and after corticosteroid therapy (*p* > 0.05), suggesting that PLR may remain a stable inflammatory marker independent of steroid exposure [27,28]. Similarly, increases in NLR in pediatric asthma have been attributed primarily to disease severity rather than corticosteroid use [29]. In contrast, the hematologic alterations observed in Duchenne Muscular Dystrophy (DMD) are more likely to reflect the underlying disease burden rather than medication effects alone. In DMD, corticosteroids are generally administered at a maintenance dose of approximately 0.75 mg/kg/day, which has been shown to slow—but not halt—disease progression. While the adverse effects of steroids are dose-dependent, their benefits in reducing muscle degeneration outweigh these risks. This benefit–risk balance may also explain why systemic inflammatory biomarkers (e.g., NLR, PLR) did not demonstrate marked abnormalities in our cohort, despite chronic steroid therapy, and should be acknowledged as a potential confounder. In addition, all patients in the study group received steroid therapy according to the same protocol, and the within-group comparisons were conducted in a clinically homogeneous patient population. Prospective studies are warranted to clarify these findings.

## 5. Conclusions

This study suggests that systemic inflammatory indices are not significantly associated with DMD. The observed correlation between NT-proBNP and PLR was unexpected and, given the small sample size, should be interpreted cautiously. Larger prospective studies are needed to clarify its significance.

## Figures and Tables

**Table 1:** Descriptive Statistics of Categorical Variables in the Patient Group (n = 25).

		N	%
Disability Status (Wheelchair Use)	Yes	4	16%
	No	21	84%
Cardiac Treatment	ACE Inhibitor (ACEI)	0	0.0%
	Beta Blocker	1	4%
	ACE Inhibitor + Beta Blocker	1	4%
	Digoxin + ACE Inhibitor + Beta Blocker	1	4%
Home Mechanical Ventilation	No	25	100.0%
Tracheostomy	No	25	100.0%

**Table 2:** Echocardiographic and Electrocardiographic Findings in the Patient Group.

Echocardiography Findings	Normal	21	84.0%
	Mitral Valve Prolapse + Mitral Regurgitation (MVP + MR)	1	4.0%
	Left Ventricular Systolic Dysfunction	1	4.0%
	Left Ventricular Systolic Dysfunction + Dilated Cardiomyopathy	1	4.0%
	Tricuspid Regurgitation	1	4.0%
Electrocardiography Findings	Pathological Q Wave	6	24.0%
	T negatifliği (İnferior Derivasyonlarda)	1	4.0%
	Normal	16	64.0%
	Right Bundle Branch Block (RBBB)	1	4.0%
	Supraventricular Tachycardia (SVT)	1	4.0%

**Table 3:** Comparison of Other Hematological Parameters Between the Groups.

	Control (n = 25)		Patient (n = 25)		
	Mean	Std. Dev.	Mean	Std. Dev.	* p
Hemoglobin	12,652	0.842	13.461	1.114	***0.006***
White Blood Cell	8278.400	2241.084	9157.826	3643.207	*0.315*
Lymphocyte	2912.400	1260.632	3200.000	1058.979	*0.399*
Neutrophil	4456.000	2316.610	5033.913	3465.274	*0.497*
Monocyte	560.400	199.885	579.565	349.876	*0.815*
Platelet	293,600.00	75,365.77	297,347.82	73,297.40	*0.862*

* Significance levels based on independent samples *t*-test results.

**Table 4:** Comparison of Inflammatory Markers Between Groups.

	Control (n = 25)		Patient (n = 25)		
	Mean	Std. Dev.	Mean	Std. Dev.	* p
NLR	2.032	1.765	2.005	2.285	*0.963*
PLR	117.609	52.916	102.636	46.921	*0.307*
MLR	0.248	0.176	0.213	0.150	*0.473*
SIRI	0.129	0.164	0.139	0.188	*0.842*
SII	588.672	560.940	592.798	659.167	*0.981*
PIV	394.378	586.885	502.8482	103.667	*0.654*

* Significance levels based on independent samples *t*-test results.

**Table 5:** Results of Correlation Analysis Between Measurements in the Patient Group.

		Pro BNP	Troponin T	CKMB	Troponin I
PLT	r	−0.152	0.453	0.237	−0.487
	*p*	0.773	0.547	0.763	0.268
NLR	r	0.599	0.142	0.445	−0.246
	*p*	0.209	0.858	0.555	0.595
PLR	r	0.860 *	−0.523	0.872	−0.510
	*p*	**0.028**	0.477	0.128	0.243
MLR	r	0.473	−0.589	0.350	−0.319
	*p*	0.343	0.411	0.650	0.486
SIRI	r	0.349	0.361	0.084	−0.229
	*p*	0.498	0.639	0.916	0.621
SII	r	0.688	0.392	0.594	−0.330
	*p*	0.131	0.608	0.406	0.470
PIV	r	0.475	0.521	0.266	−0.261
	*p*	0.341	0.479	0.734	0.572
	*p*	0.188	0.254	0.129	0.545

r: Pearson correlation coefficients; *: *p* < 0.05.

**Table 6:** ROC Analysis of Measurements Across Groups.

Test Variables	Area (AUC)	Std. Error	*p*	Cut-Off	Sensitivity	Specificity
NLR	0.565	0.085	*0.439*	1.21	0.568	0.600
PLR	0.591	0.083	*0.279*	98.15	0.565	0.560
MLR	0.575	0.084	*0.375*	0.17	0.522	0.520
SIRI	0.572	0.085	*0.392*	0.611	0.527	0.522
SII	0.534	0.085	*0.687*	386.284	0.525	0.526
PIV	0.565	0.085	*0.439*	191.807	0.609	0.605

AUC: Area Under Curve. Categorical variable: Group Cut-Off: Cut-off value.

**Table 7:** Comparison of Measurements According to EKG/ECHO Abnormalities.

	EKG/ECHO Abnormality				
	No (n = 15)		Yes (n = 10)		* p
	Mean	Std. Dev.	Mean	Std. Dev.	
Age	10.733	4.334	12.100	4.483	*0.454*
Age at Diagnosis	4.667	2.498	5.100	2.998	*0.698*
Hemoglobin	13.146	1.182	13.870	0.918	*0.125*
WBC	8419.231	2758.380	10,118.00	4526.342	*0.278*
Lymphocyte	3352.308	1145.136	3002.00	956.960	*0.444*
Neutrophil	4181.538	2754.499	6142.00	4101.598	*0.185*
Monocyte	598.462	391.490	555.00	306.095	*0.775*
PLT	291,538.46	74,891.72	304,900.00	74,446.77	*0.675*
NLR	1.705	2.415	2.395	2.166	*0.485*
PLR	97.195	51.153	109.709	42.360	*0.539*
MLR	0.212	0.144	0.215	0.167	*0.965*
SIRI	0.114	0.158	0.174	0.227	*0.461*
SII	502.861	717.859	709.717	590.039	*0.468*
PIV	519.283	131.074	481.482	579.162	*0.933*
Glucose	94.167	11.846	99.100	16.381	*0.422*
Creatinine	0.277	0.113	0.250	0.164	*0.658*
Aspartate Aminotransferase (AST)	166.083	151.280	148.500	115.591	*0.766*
Alanine Aminotransferase (ALT)	191.417	184.558	129.500	117.995	*0.371*
Alkaline Phosphatase (ALP)	145.000	21.714	118.200	66.744	*0.418*
Gamma-Glutamyl Transferase (GGT)	16.250	18.661	6.000		*0.657*
Bilirubin	0.574	0.351	0.572	0.235	*0.987*
Creatine Kinase (CK)	7608.889	5705.158	3734.833	2767,020	*0.149*
ProBNP	16.900		295.240	405.682	*0.565*
TroponinT	101.000		127.167	33.773	*0.571*
Troponin I	0.215	0.163	0.106	0.009	*0.135*

* Significance levels based on independent samples *t*-test results.

## Data Availability

The original contributions presented in the study are included in the article, further inquiries can be directed to the corresponding author.

## Abbreviations

The following abbreviations are used in this manuscript:

DMD	Duchenne Muscular Dystrophy
NLR	Neutrophil-to-Lymphocyte Ratio
PLR	Platelet-to-Lymphocyte Ratio
MLR	Monocyte-to-Lymphocyte Ratio
SII	Systemic Immune-Inflammation Index
SIRI	Systemic Inflammatory Response Index
PIV	Pan-Immune Inflammation Value
ECG	Electrocardiography
Pro-BNP	Pro-Brain Natriuretic Peptide
ROC	Receiver Operating Characteristic
ECHO	Echocardiography
SPSS	Statistical Package for the Social Sciences
AUC	Area Under the Curve
ACE	Angiotensin-Converting Enzyme
TTE	Transthoracic Echocardiography
RBB	Right Bundle Branch
SVT	Supraventricular Tachycardia
WBC	White Blood Cell
PLT	Platelet
CK-MB	Creatine Kinase-MB Isoenzyme
AST	Aspartate Aminotransferase
ALT	Alanine Aminotransferase
ALP	Alkaline Phosphatase
GGT	Gamma-Glutamyl Transferase (Gama-Glutamil Transferaz)
CK	Creatine Kinase
SCD	Sudden Cardiac Death
